# Chest Wall Mass in Infancy: The Presentation of Bone-Tumor-Like BCG Osteitis

**DOI:** 10.1155/2020/8884770

**Published:** 2020-11-11

**Authors:** Phumin Chaweephisal, Teesit Torchareon, Shanop Shuangshoti, Piti Techavichit

**Affiliations:** ^1^STAR Pediatric Hematology and Oncology, Department of Pediatrics, Faculty of Medicine, Chulalongkorn University, Bangkok, Thailand; ^2^Department of Pediatrics, Vachira Phuket Hospital, Phuket, Thailand; ^3^Departments of Pathology, Faculty of Medicine, Chulalongkorn University, Bangkok, Thailand

## Abstract

Chest wall mass in infancy is rare. Malignant lesions are more common than infection or benign tumors. This is a case of a 12-month-old girl who presented with a 2 cm mass at the right costal margin and poor weight gain. Chest radiograph demonstrated a moth-eaten osteolytic lesion at the 8th rib. The resection was performed, and a mass with pus content was found. The positive acid fast stain (AFB) organism was noted. Pathology confirmed caseous granulomatous inflammation compatible with mycobacterial infection. However, QuantiFERON-TB Gold was negative, so *Mycobacterium bovis* (*M. bovis*) osteitis is highly suspected. She was treated with antimycobacterium drugs and showed good results. Osteomyelitis can manifest by mimicking bone tumors. Without a biopsy, the pathogen may go undetected. So, interventions such as biopsy are warranted and avoid mass resection without indication. High C-reactive protein (CRP), alkaline phosphatase (ALP), periosteal reaction of radiating spicules, and penumbra sign in magnetic resonance imaging (MRI) are helpful for discriminating osteomyelitis from bone tumor.

## 1. Introduction

Chest wall mass in infancy is rare [1]. As an infectious cause, benign and malignant tumors must be a concern. However, malignant lesions are more common than the others [2] and favor metastasis [3]. A primary neoplasm of the thoracic wall is caused only in 5–10% [4] of bone tumors. Malignancies include Ewing's sarcoma, Langerhans cell histiocytosis, osteosarcoma, primitive neuroectodermal tumor, metastatic neuroblastoma, and leukemia [5].

Infection in the form of chest wall osteomyelitis can be caused by bacteria, fungi, and mycobacteria, whether from direct extension of empyema and pneumonia or hematogenous spread [5]. World incidence of new tuberculosis (TB) is about 142 per 100,000 population reported each year [6], with 29% cases being extrapulmonary. Bones and joints are affected by only 1% infection of all TB manifestations [7].

## 2. Case

A 12-month-old girl came from Nakornsawan province, who presented with painless progressive palpable round shape mass at the right costal margin. She had no fever. Her appetite was good, but still she had poor weight gain with height and weight both lower than fifth percentile. No history of contact with tuberculosis was found, but her mother had a positive history of TB lymphadenitis and completed treatment more than ten years ago. The natal history was unremarkable. She completed the Thai national immunization program and was given complementary vaccines including pneumococcus, *H. influenza*, and intradermal tuberculosis vaccine at birth.

**Figure 1:**
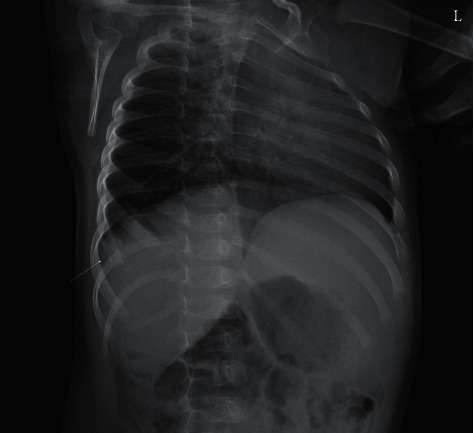
Chest radiograph demonstrated moth-eaten osteolytic lesion at the right anterior 8^th^ rib.

**Figure 2:**
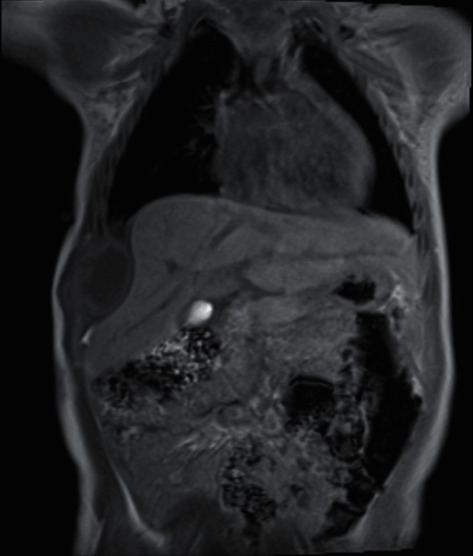
Chest MRI shows lobulated irregular rim-enhancing lesion epicenter with destruction of the right lateral 8^th^ rib causing pressure effect to right hepatic lobe. Penumbra sign is positive.

Physical examination revealed a 2 cm round fixed firm mass at the right anterior lower chest wall. Bacillus Calmette–Guérin (BCG) scar was found at right upper leg. Otherwise, it was unremarkable. Chest radiograph demonstrated a moth-eaten osteolytic lesion at the right anterior 8^th^ rib, shown in Figure 1. Complete blood count showed the following: hemoglobin: 11.2 (>10.5) g/dL, white blood cell: 12,790 (6,000–17,500)/mcL (*N*: 20.4%, *L*: 73.1%), PLT: 339,000/mcL, alkaline phosphatase: 201 (150–420) U/L, and hs-CRP: 20.98 (0–5) mg/L. Chest magnetic resonance imaging (MRI) is shown in Figure 2. The presumptive diagnosis is probable Ewing's sarcoma or Langerhans cell histiocytosis.

**Figure 3:**
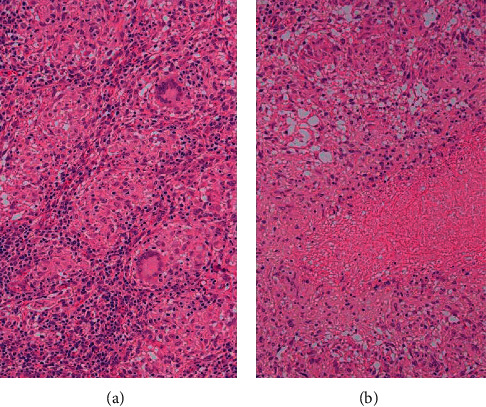
Microscopic examination showed granulomatous inflammation with caseous necrosis.

Resection of the mass was arranged. Intraoperative examination revealed a firm round 5 cm mass with signs of osteomyelitis on the rib and pus. The pus was further processed and revealed positive acid fast bacilli stain (AFB) and was reactive of polymerase chain reaction (PCR) for *Mycobacterium tuberculosis* complex (MBTC). Pathology, shown in Figure 3, confirmed caseous granulomatous inflammation compatible with mycobacterial infection. A positive purified protein derivative (PPD) skin test at 72 hours was reported afterward. However, mycobacterial pus culture, QuantiFERON-TB Gold, 3-day morning gastric content for AFB, MTBC PCR, and culture were all negative. Because of clinical and negative QuantiFERON-TB Gold, *Mycobacterium bovis* (*M. bovis*) osteitis is highly suspected. She was treated with HRZE standard regimen (isoniazid, rifampicin, pyrazinamide, and ethambutol) for 2 months and switched to HR (isoniazid and rifampicin) for a total of 12 months. Outpatient 3-year follow-up demonstrates excellent weight gain, good appetite, and normal wound healing without mass progression.

## 3. Discussion

The diagnostic guidelines recommend that the diagnosis of TB should follow the cautious history of TB contact, symptoms and signs, abnormal chest radiograph, sputum/gastric aspiration smear AFB/culture, and tuberculin skin test [7]. Negative QuantiFERON-TB Gold reflects that the organism may not be *M. tuberculosis*. Therefore, other species should be suspected including *M. bovis.*

Bacillus Calmette–Guérin (BCG), Tokyo 172-strains, was employed for preventing tuberculosis and has shown excellent results in preventing miliary and meningeal tuberculosis [8]. Although the report of Tokyo 172-strains vaccine in Taiwan demonstrated that incidence of BCG osteitis/osteomyelitis was 30.1 cases per million vaccinations during years 2008–2012. *M. bovis* infection is more likely to present with extrapulmonary infection [9, 10]. Osteomyelitis commonly involves the long bone of extremities with 80% located on epiphysis or metaphysis [11]. Only 2% occurrence of tuberculosis in rib was reported in the southeast Asian endemic area [12].

Review from 8 cases of chest wall mycobacterial infection (Table 1) shows that the male-to-female ratio is 1 : 1. The age range was from 9 months till 10 years (median age: 13.5 months). Pathogens vary from *M. bovis* to *M. tuberculosis*. Infected sites are often the rib (6 of 8 cases), and the others are sternum, chest wall, and pulmonary and hematologic dissemination. *M. bovis* infection was only found in the infant period. Therefore, if age is more than 12 months, *M. tuberculosis* should be considered first [9, 12–18].

Naturally, *M. bovis* is resistant to pyrazinamide. So, the combination of 2HRZE + 10HR is strongly recommended for osteomyelitis and a longer course is considered in severe disease [8].

Osteomyelitis can manifest by mimicking bone tumors. The most common pathogen in nontuberculous endemic areas are bacteria such as *Staphylococcus aureus* and *Klebsiella pneumoniae* [19]. Without a mass biopsy, the pathogen may go undetected. So, interventions such as biopsy are warranted. Mass resection with bone debridement will avoid the need for and delay caused by multiple trials of antimicrobial agents. So, the infection always occurs in the differential part of chest wall mass, and only biopsy is required. Even incision and drainage should be avoided in BCG osteitis due to the possibility of delayed wound healing. However, the needle tract should be appropriately placed to be able to subsequently perform en-bloc resection in case the biopsy reveals a malignant pathology.

It is not possible to distinguish between osteomyelitis and a bone tumor clinically. The majority of osteomyelitis cases did not show concurrent fever and mild or no local reactions [20]. But multiple methods of laboratory and imaging techniques are proposed in Table 2. High C-reactive protein (CRP) shows sensitivity of 60% and specificity of 90.8% [22] for diagnosis of osteomyelitis. The levels of calcium, phosphate, and alkaline phosphatase are unremarkable in osteomyelitis, in contrast to metastatic or some metabolic bone diseases [23]. The white blood cell count in some reports fails to show a correlation [22]. Pathological fractures are often reported in malignant bone tumors and extremities are also reported in osteomyelitis [22]. Plain radiographs fail to distinguish these two disease entities since the lytic and sclerotic sign can be found in both. But in osteomyelitis, most periosteal reactions do not report radiating spicules which differ from osteosarcoma and Ewing's sarcoma. Penumbra sign in T1-weighted magnetic resonance (MR) images has also been reported to be helpful for discriminating osteomyelitis with a sensitivity of 73.3% and specificity of 99.1% [22]. This sign is characterized by a ‘‘target” appearance with four layers. The hypointense central abscess cavity is followed by an inner relatively hyperintense ring of granulation tissue, an outer hypointense ring, and a peripheral hypointense halo. However, tumors with internal bleeding may cause false positive results, and acute osteomyelitis may cause a false negative sign.

## 4. Summary

This case report shows an atypical finding of a rib mass in infancy. As atypical infection, benign and malignant tumors must be considered. Although infection is in the differential, tissue biopsy or resection may be necessary. CRP, ALP, and penumbra sign on MRI may support the diagnosis of osteomyelitis and favor a biopsy rather than mass excision. For younger patients with mycobacterium osteomyelitis or abscess, *M. bovis* should be proven due to difference in drug regimens.

## Figures and Tables

**Table 1:** Review reports from 8 cases of chest wall mycobacterial infection.

No.	Year	Patient profile	Site	Work up	Organism
1 [13]	2001	9 months, male, Turkey	Parasternal abscess with sternum osteomyelitis	Tissue pathology	Suspected *M. bovis*
2 [14]	1992	11 months, male, German	Mass with 4^th^ rib osteomyelitis	Tissue pathology	*M. bovis* isolated
3 [9]	2005	12 months, female, Taiwan	Subcutaneous abscess, left anterior chest wall	Bacterial isolation	*M. bovis* isolated
4	2017	12 months, female, Thailand	8^th^ rib osteomyelitis	Tissue pathology	Suspected *M. bovis*
5 [15]	2012	15 months, female, Italy	Mass with 10th rib osteomyelitis, pulmonary	Tissue pathology	*M. tuberculosis* isolated
6 [12]	2001	4-year-old, female, Taiwan	Chest wall abscess and third rib osteomyelitis/disseminated TB	Tissue pathology	Suspected *M. tuberculosis*
7 [16]	2006	9-year-old, male, India	Abscess, 2^nd^ rib osteomyelitis	Aspiration	Suspected *M. tuberculosis*
8 [17]	2017	10-year-old, female, India	Chest wall abscess and 5^th^ rib osteomyelitis	Aspiration	Suspected *M. tuberculosis*

**Table 2:** Comparison of clinical characteristics of bone tumors and osteomyelitis of the chest wall.

	Bone tumor	Osteomyelitis of chest wall
Cause	Malignant: Ewing sarcoma, Langerhans cell histiocytosis, osteosarcoma, primitive neuroectodermal tumor, metastatic neuroblastoma and leukema Benign: fibrous dysplasia, enchondroma, osteochondroma, etc. [21]	Common: *Staphylococcus aureus, Klebsiella pneumoniae* Rare: propionibacterium, mycobacterial species, salmonella, etc.
Median time to diagnosis	Vary among tumor behavior and pathology.	Median time 5.4 months (ranged 2 weeks to 1 years) [19]
Alkaline phosphatase (ALP)	Elevated or normal	Normal
C-reactive protein (CRP)	Normal	Elevated
WBC count	Normal	Rarely elevated
Plain radiologic finding	Osteosclerotic, lytic lesion depend on tumor type	Lytic lesion is more common than osteosclerosis [19]
Periosteal reaction	Lamellated periosteal reaction, radiating spicules (sunburst)	Lamellated periosteal reaction
Penumbra sign on T1W MRI	Rare	Occasional
Surgery	Resection is required in most	Biopsy is required in most; debridement is limited for refractory cases

## Data Availability

The data used to support the findings of this study are included within the article.
